# Distributed Destination Search Routing for 5G and beyond Networks

**DOI:** 10.3390/s22020472

**Published:** 2022-01-08

**Authors:** Abdullah Waqas, Nasir Saeed, Hasan Mahmood, Muhannad Almutiry

**Affiliations:** 1 Department of Electrical Engineering, National University of Technology, Islamabad 44000, Pakistan; nasirsaeed@nutech.edu.pk; 2 Department of Electronics, Quaid-I-Azam University, Islamabad 44000, Pakistan; hasan@qau.edu.pk; 3 Department of Electrical Engineering, Northern Border University, Arar 73222, Saudi Arabia; muhannad.almutiry@nbu.edu.sa; 4 Remote Sensing Unit, Northern Border University, Arar 73222, Saudi Arabia

**Keywords:** 5G and beyond networks, energy efficient, routing, AODV

## Abstract

Fifth-generation and beyond networks target multiple distributed network application such as Internet of Things (IoT), connected robotics, and massive Machine Type Communication (mMTC). In the absence of a central management unit, the device need to search and establish a route towards the destination before initializing data transmission. In this paper, we proposes a destination search and routing method for distributed 5G and beyond networks. In the proposed method, the source node makes multiple attempts to search for a route towards the destination by expanding disk-like patterns originating from the source node. The source node increases the search area in each attempt, accommodating more nodes in the search process. As a result, the probability of finding the destination increases, which reduces energy consumption and time delay of routing. We propose three variants of routing for high, medium, and low-density network scenarios and analyze their performance for various network configurations. The results demonstrate that the performance of the proposed solution is better than previously proposed techniques in terms of time latency and reduced energy consumption, making it applicable for 5G and beyond networks.

## 1. Introduction

Fifth-generation (5G) and beyond networks aim to provide users with high data rates, full network coverage, high connectivity, and improved security. Therefore, 5G and beyond networks enable various new applications such as connected robotics, virtual reality, augmented reality, and Wireless Brain–Computer Interaction (WBCI). Nevertheless, the existing networking techniques in 5G systems face many challenges related to enabling these applications. For instance, the conventional infrastructure-based networks cannot fulfill high data rates, full network coverage, and high connectivity requirements due to their high cost of infrastructure installment and static configuration. Therefore, the requirement of a self-organizing and self-sustainable network inherently emerges when developing 5G technologies. Moreover, these networks also require reduced energy consumption to enhance battery lifetime inherited in the design. Various methods exist in the literature that addresses the energy consumption issue for 5G and beyond networks. For example, ref. [1] suggests that energy harvesting and energy transfer will be major components of 5G and 6G systems to prolong the lifetime of the energy-constrained devices. Furthermore, ref. [2] provides a detailed study of different energy harvesting techniques for 5G and beyond systems. In [3], the authors improve energy efficiency by using a secondary user trading method for spectrum sharing. In [4], the authors propose a Multivariate Regressive Deep Stochastic Artificial Structure Learning (MRDSASL) algorithm that improves energy consumption on the minimum cost by selecting the node with higher energy, signal strength, and spectrum utilization.

Since the architecture of 5G and beyond networks is distributed in nature with high-performance requirements, routing algorithms should have low latency, low energy consumption, and high throughput [5]. For instance, Ad Hoc Demand Distance Vector (AODV) routing protocol developed for distributed networks by Internet Engineering Task Force (IETF) can be modified for 5G and beyond networks with improved performance. In AODV, the source node searches a route towards the destination before initiating data packets [6]. The source node broadcasts a route request (RREQ) packet in the network during route search. The destination node receives the request and acknowledges a route reply (RREP) packet via a reverse path to establish a communication link. AODV uses Expanding Ring Search (ERS) technique to discover the route towards the destination [7]. In ERS, the source node makes multiple attempts to locate the destination in the network and increases the search area if a search attempt fails [8]. The search cost of ERS is directly proportional to the number of attempts made by the source during the destination search process.

The unique idea of ERS gained the attention of researchers due to its reduced energy cost. Therefore, many studied has been proposed to improve the performance of ERS routing. For instance, in [9], the authors use ERS to perform service discovery for multiple servers in Mobile Ad Hoc Networks (MANETs). In [10], the authors discuss the dependence of the initial value of TTL on energy consumption. The authors in [11] introduce a blocking procedure to reduce the cost of ERS. However, the idea of increased waiting time in blocking ERS adds time latency during route discovery. Similarly, ref. [12] modifies ERS for secure and energy efficient routing against black-hole attacks in [12].

While the development of 5G protocols is in process, researchers are studying the feasibility of modifying available 4G protocols for application on 5G. For instance, in [13], the authors used a modified version of AODV for routing in swarm Unmanned Aerial System (UAS). The modified version uses ant colony optimization technique for beam-forming and reduces routing overhead, improves throughput, and reduces time latency to meet existing 5G requirements.

In this paper, we propose Expanding Disk Search (EDS) algorithm for destination search in 5G and beyond networks. EDS algorithm searches the destination in the form of expanding disk-like patterns that increase destination search probability. The process reduces the number of attempts required by the source node to search the destination; as a result, it improves energy consumption and reduces time latency of the route. We show the efficiency and effectiveness of the proposed solution both analytically and in simulations. The results are generated for various network scenarios that show that EDS performs better than conventional route discovery techniques. Therefore, EDS is more feasible to apply in 5G and beyond networks where minimum time latency and reduced energy consumption are required. The analysis shows that the proposed technique is suitable for diverse network applications that require less energy consumption and time to discover the route. The contributions of the paper are summarized as follows:

1. Firstly, we introduce a novel scheme for TTL criteria, i.e., TTL field can take any value such as energy, interference, and time instead of limiting it to hop count.
2. Secondly, we propose a circular disk-shaped region instead of rings for route search for improved performance. The hop count-based TTL logic is replaced with real-time stamping using timers.
3. Thirdly, we modify the process of RREQ with new TTL field. In this manner, we address diverse network configurations with three different methods to increment the TTL value.
4. Finally, the proposed EDS algorithm is integrated into AODV, and performance is analyzed in terms of throughput and time latency.

The rest of the paper is organized as follows. In Section 2, we present an analysis of the previously proposed route discovery solutions for various network applications. In Section 3, we present the proposed technique and provide a comprehensive analysis to show the effectiveness of our solution. The section also presents various methods that can be used to increment TTL at intermediate nodes. In Section 4, we present the simulation environment and results for various network scenarios. In Section 5, we conclude the work.

## 2. Related Work

Routing algorithms proposed for distributed networks can be divided into three major categories: proactive routing, reactive routing, and hybrid routing. Proactive routing protocols periodically update routing tables to ensure that routing information is available whenever required without any delay. However, this periodic update of routing tables introduces traffic overhead, increasing the number of nodes in the network. In addition, the proactive protocols are not suitable for networks with highly dynamic network configuration; therefore, these protocols are not suitable for application in 5G and beyond networks where the network is dynamic. Hybrid routing protocols face a similar problem when applied in scenarios where the nodes are mobile, and link formation changes frequently. In contrast to the protocols mentioned above, reactive routing protocols establish routes on demand.

There are many reactive routing protocols in the literature to search a route within the network area [14,15,16]. The conventional approach is to locate the destination with simple flooding, in which the source node broadcasts the RREQ packet in the entire network. The blind nodes rebroadcasting RREQ packets result in *broadcast storm*, which introduces high overhead, collision, and congestion in the network [17]. A solution to handle the broadcast storm problem in reactive routing protocols is using probabilistic broadcasting [18]. In this method, each node transmits the packet with some probability $P_{i}$ and drops the packet with probability $1-P_{i}$. The value of $P_{i}$ is a design parameter that can be selected according to the application requirement. However, the probabilistic broadcasting method encounters a problem that some nodes may not receive RREQ packets. The Connected Dominating Set (CDS) is another scheme to control redundant broadcast during route discovery [19]. Forming CDS enhances the packet delivery ratio, and the solution works well for a highly dynamic environment. Another scheme presented in [20] is to handle broadcast storms by a grid-based mechanism. In this scheme, the network is partitioned into grids, and each node is responsible for broadcasting the packet in its own grid. Both the CDS scheme and grid forwarding scheme require knowledge of network topology or some information about the location of network nodes, adding hardware cost such as having a GPS receiver at each node.

In [21], the authors proposed Expanding Ring Search (ERS) to reduce the number of RREQ packets relayed in the network during the route discovery process in wireless multihop networks. The area of the ring is increased if the destination is not found in the previous attempt, optimizing the number of attempts required to minimize the energy consumption. In [22], the authors provide another method to improve energy consumption of ERS by using information available after the first attempt, showing improvement of up to 20%. In [10], the authors proposed a search set to optimize the expanding ring search algorithm. In [23], the authors proposed another scheme to improve further the energy consumption of ERS, which is named Blocking ERS. However, the time latency of Blocking ERS is higher than ERS. The performance of ERS is highly dependant on the choice of the threshold value of TTL that depends on network parameters such as the number of nodes, network density, and network topology. Table 1 summarises previously proposed routing methods and their limitations. In the subsequent section, we discuss the proposed routing algorithm in detail.

## 3. Proposed Expanding Disk Search Algorithm

**Figure 1:**
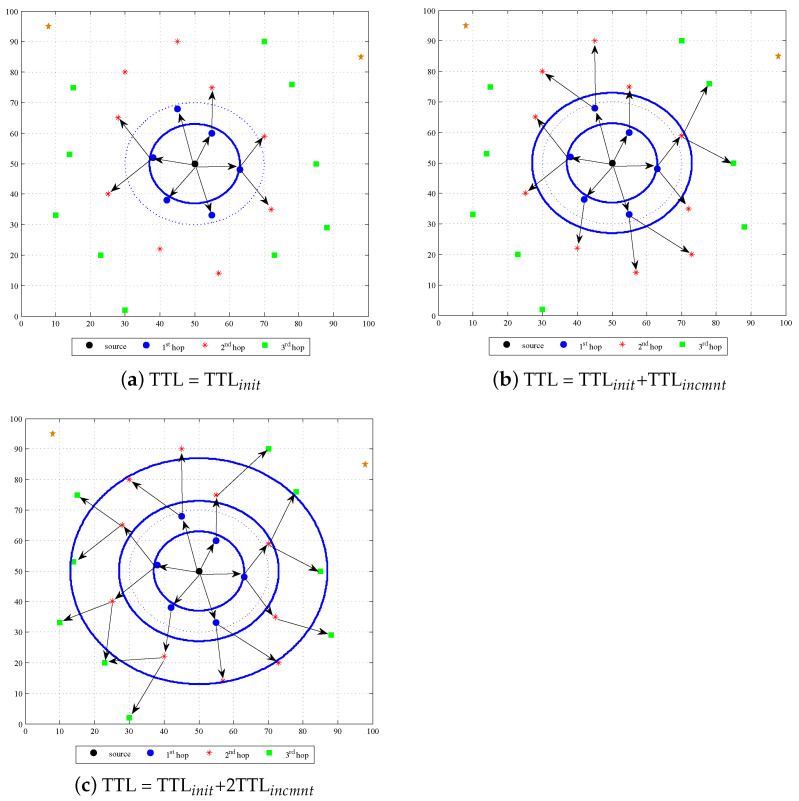
Forwarding of RREQ in EDS.

**Figure 2:**
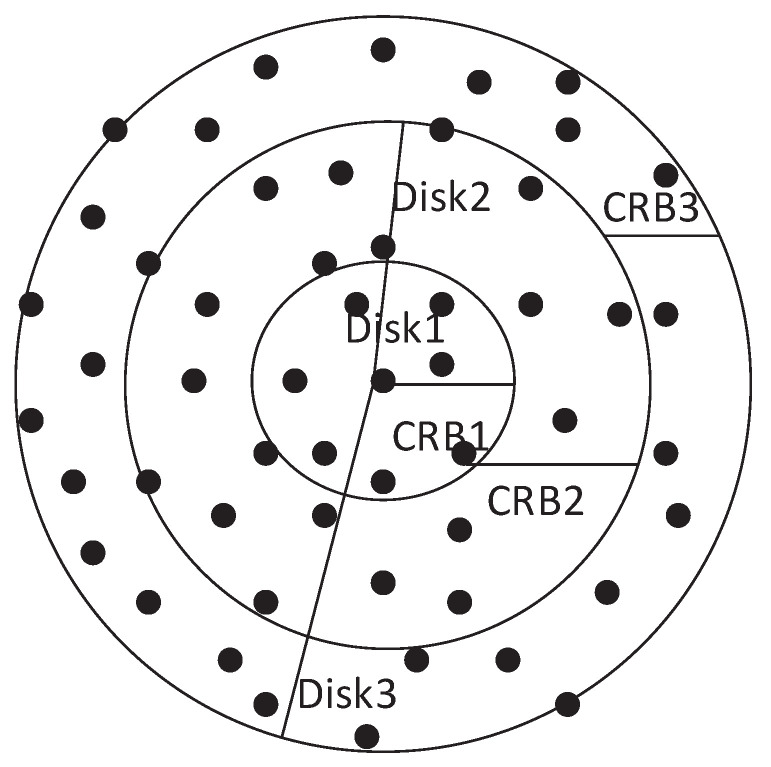
Formation of CRB and Disks in EDS.

We propose a novel routing technique named Expanding Disk Search (EDS) in distributed networks. The conventional routing methods use hop count as TTL value in the header of RREQ packet to limit the transmission of RREQ packet in the entire network. However, the methods may result in high time latency in situations where multiple sources are transmitting to multiple destinations simultaneously due to high traffic load at intermediate nodes, which results in long queues. Therefore, in contrast to conventional search methods, we used time stamp as TTL value in the header of RREQ packet to calculate the expiry time of the route search. When a source node wants to establish a communication link with a destination, it initiates the search query in the network with an initial time stamp. A new search query with a higher expiry time value is initiated if the previous search fails to locate the destination. As a result, the area of the search disk increases with each search attempt, as shown in Figure 1. Since the disks accommodate more nodes than rings, the probability of finding the destination in a search increases, and the search time reduces. We call the area between adjacent disks as Circular Ring Band (CRB), which is shown as CRB1, CRB2, and CRB3 in Figure 2. The area of each disk can be controlled by adjusting the time stamp value in the TTL field of the RREQ packet. From an implementation point of view, EDS requires that the clock of the nodes is synchronized to correctly calculate the expiry of the RREQ packet. During practical implementation, an efficient clock synchronization mechanism can be implemented at each node to achieve synchronization [26,27,28]. While clock synchronization in distributed network is not the focus of this article, we assume that the clock of each node is synchronized. The area of each disk and CRB is influenced by the choice of the TTL value that can be calculated as given in Table 2.

**Figure 3:**
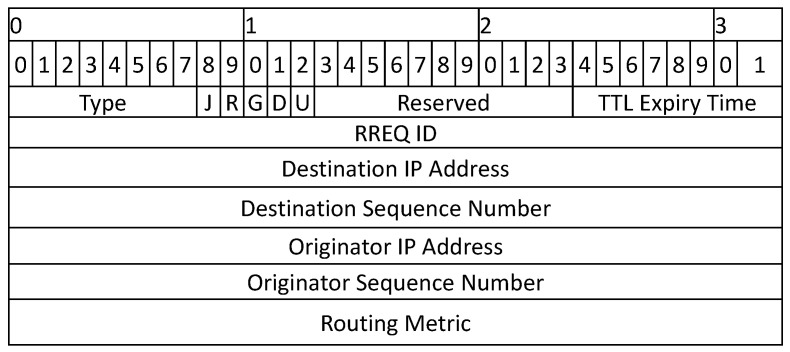
Packet Formats for RREQ.

**Figure 4:**
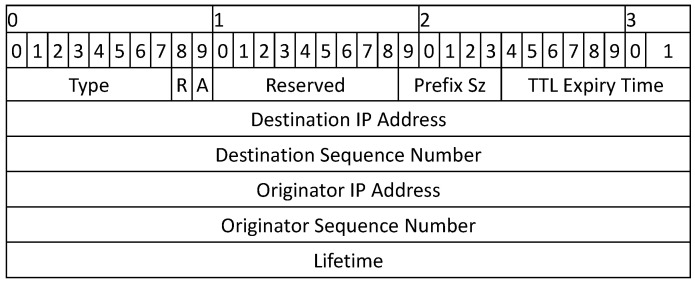
Packet Formats for RREP.

When the source node desires communication with a destination, it initiates a route discovery process and broadcasts an RREQ packet to its neighbors. The format of the new RREQ packet and RREP packets is shown in Figure 3 and Figure 4, respectively. The RREQ packet has a time stamp, which is used to estimate the travel time of a packet. When a node receives the RREQ packet, it responds with an RREP packet if it is the destination; otherwise, it compares the time stamp in the RREQ packet with its local time and drops the packet if the packet is expired. If the packet is not expired, the relay node forwards the packet to its neighbors, and the process continues until the packet reaches the destination. If the source node does not find the destination in the first attempt, it searches the large area in the next try with an incremental expiry time value. The source node waits for a predefined time to receive RREP from its neighbors before initiating the next attempt. The *waiting time* is normally a constant value and is assumed to be half of the TTL value [29]. The value of expiry time is directly proportional to search attempt and can be calculated as follows:

(1) $$TTL_{expiry}=t_{s}+f\left(t_{i}\right)\times t_{s},$$

where $t_{s}$ represents the local time of the source node, and $t_{i}$ is the incremented value of TTL in the attempt. The performance of the proposed scheme depends on the choice of parameter $t_{i}$. In the proceeding sections, we will discuss in detail various methods that can be used to choose the appropriate value of $t_{i}$ for various network scenarios. During the search process, the source node increments the TTL values until a threshold is reached. If the destination is not found within the threshold number of attempts, the source node broadcasts the packet in the entire network. The broadcast packet has a relatively high value of TTL as compared to RREQ search packets. Algorithm 1 demonstrates the step-by-step procedure of the EDS technique.
**Algorithm 1:** Pseudocode for EDS**if** source node **then**   $t_{s}$ = get current time;   number of attempts i = 1;   TTL = $t_{1}$ + $t_{s}$;   **while** TTL < TTL −thredhold **do**     broadcast RREQ packet with TTL value;     start waiting timer $t_{w}$ with value = T;     **if** RREP received **then**        start sending data;        break;     **end if**     **if** $t_{w}$ reaches 0 **then**        $t_{s}$ = get current time;        i = i + 1;        **if** technique = EDS-S **then**          $t_{1}$ = $\sqrt{i}t_{1}$ + $t_{s}$;        **end if**        **if** technique = EDS-L **then**          $t_{1}$ = i $t_{1}$ + $t_{s}$;        **end if**        **if** technique = EDS-R **then**          $t_{1}$ = $\sqrt{\frac{i(i+1)}{2}}$$t_{1}$ + $t_{s}$;        **end if**        TTL = $t_{1}$ + $t_{s}$        broadcast RREQ packet with TTL value;        start waiting time $t_{w}$ with value = i × T;     **end if**   **end while**   broadcast RREQ with TTL = $TTL_{max}$;**end if****if** relay node **then**   **if** receive a RREQ **then**     **if** has fresh route information **then**        send the RREP towards source node;     **else**        **if** TTL ≤ local time **then**          forward RREQ;        **else**          drop RREQ;        **end if**     **end if**   **end if****end if****if** destination node **then**   wait for RREQ packet   **if** receive first RREQ **then**     send RREP towards source;     save metric value;   **else**     **if** metric value < saved metric value **then**        send RREP towards source;        save new metric value;     **else**        drop the packet;     **end if**   **end if****end if**

### 3.1. Destination Discovery Time

In the absence of location information of the destination node, the source node makes multiple attempts to locate the destination within the network region. In this process, the source node waits for a predefined time after a request query is broadcast in the network. We defined the *Disk Access Time* as the time taken by the packet to access the particular disk taking the discovery start time as a reference. As shown in Table 3, the time to locate the destination in a disk is directly proportional to the number of attempts made by the source node. In addition, it also depends on the probability of finding the destination in a disk. The time to establish a route is the sum of time required to locate the destination, time to receive the RREP packet, and the disk access time, which is given as follows:

(2) $$E\left(t\right)=\sum_{i=1}^{L}t_{i}P_{ci}+\sum_{i=1}^{L}P_{ci}T,$$

where $P_{ci}$ is the probability of whether the destination lies in the *i*th CRB, $t_{i}$ is the TTL value required to search the *i*th disk, *T* is the time after which next incremental search is initiated, and *L* is the threshold value of the TTL after which the source node decides to broadcast the packet. The expression in (2) shows that the expected time to find the destination depends on the probability to find the destination in a disk, the choice of TTL value, the interval between two successive attempts, and the choice of threshold value.

### 3.2. Energy Consumption

The energy consumption of EDS algorithm is measured in terms of number of RREQ packets relayed in the network. Energy consumption of EDS can be calculated as follows [30]:

(3) β(L)=X(L)+Z(L)Y(L),

where the following is the case:

(4) $$X\left(L\right)=\sum_{i=1}^{L}\left(P_{ci}\sum_{k=1}^{i}\left[1+\sum_{j=1}^{k-1}n_{j}\right]\right),$$

(5) $$Y\left(L\right)=\sum_{k=1}^{L}\left(1+\sum_{j=1}^{k-1}n_{j}\right)+N-1,$$

and the following is obtained.

(6) $$Z\left(L\right)=1-\sum_{i=1}^{L}P_{ci}.$$

Here, $n_{j}$ is the number of nodes in *j*th CRB, and $P_{ci}$ is the probability that the destination lies in the *i*th CRB. The values $n_{j}$ and $P_{ci}$ depend on the choice of TTL value. The expressions in (3)–(6) represents the Expected Broadcast Cost (EBC) of EDS algorithm as a function of number of attempts required to search the destination. In (4), the summation variable *k* accumulates the number of packets transmitted by each node in one search operation. Variable *i* varies from 1 up to *L* to collect the broadcast cost of each search query during the EDS process. The expression in (5) represents the cost of the search operation if the destination is not found within the *L* number of attempts. The expression in (6) represents the probability that the destination is not found within the *L* number of attempts. The TTL value required to search the *i*th disk is a function of the initial value of TTL. In the following sections, we proposed three variants of the EDS algorithm that can be used for energy-efficient route search in distributed networks.

### 3.3. Increment TTL with Square Root Function (EDS-S)

**Figure 5:**
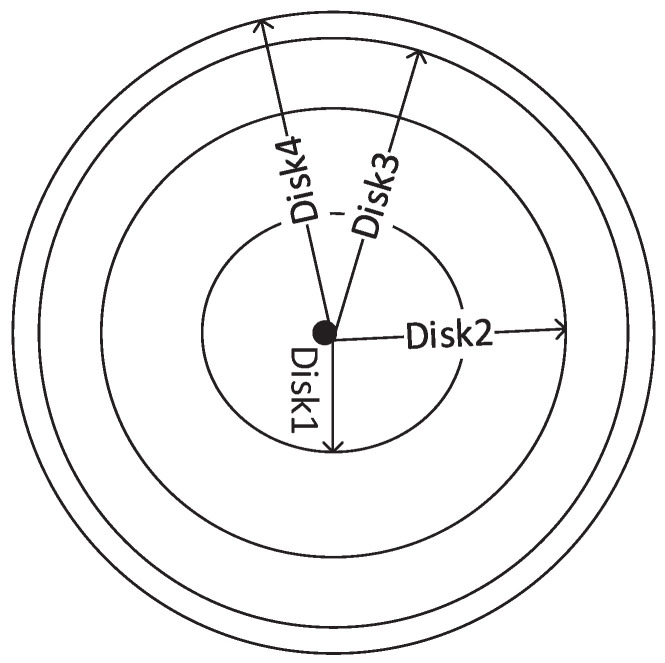
Disks Formation for EDS-S.

In this section, we derive the expression for search time and energy consumption when TTL is varied in such a manner that the area of each CRB remains the same, as shown in Figure 5. Mathematically, $A_{c1}=A_{c2}=A_{c3}=\ldots =A_{cn}$, where $A_{ci}$ is the area of ith CRB. If $t_{i}$ is the TTL value required to search the area of radius $r_{i}$, then $A_{di}=\pi t_{i}^{2}$, $A_{ci}=A_{di}-A_{d(i-1)}$, and $A_{di}=\pi \left(t_{i}^{2}-t_{i-1}^{2}\right)$. Let us assume that $A_{d0}$ is the area of disk0 ($A_{d0}=0$) that contains the source node only. As the area of each CRB is the same, then $A_{c2}=A_{c1}$, $\pi \left(t_{2}^{2}-t_{1}^{2}\right)=\pi \left(t_{1}^{2}-t_{0}^{2}\right)$, and $t_{2}^{2}=2t_{1}^{2}-t_{0}^{2}$, where $t_{0}$ is the TTL value required to search disk0 ($t_{0}=0$), which contains the source node only. Therefore, $t_{2}=\sqrt{2}t_{1}$ and $t_{3}=\sqrt{3}t_{1}$. In general, we have the following.

(7) $$t_{i}=\sqrt{i}t_{1}.$$

The expression in (7) provides the formula required to search the *i*th disk as a function of TTL value. Moreover, we assume that *N* nodes are uniformly distributed in the network area $x^{2}$. As the area of each CRB is the same, the number of nodes in each CRB will be same and can be calculated as follows.

(8) $$n_{i}=\frac{\left(N-1\right)\pi t_{1}^{2}}{x^{2}}.$$

The probability to find the destination in *i*th CRB is $P_{ci}=\frac{\pi t_{1}^{2}}{x^{2}}$. The probability to find the destination in the *i*th disk is sum of probabilities up to *i*th CRB.

(9) $$P_{di}=\sum_{k=1}^{i}P_{ck}.$$

The expected time to find the destination can be calculated by placing values from (7) into (2), which yields the following.

(10) $$E\left(t\right)=\frac{2\pi t_{1}^{3}}{x^{2}}\sum_{i=1}^{L}\sqrt{i}+\frac{T\pi t_{1}^{2}}{x^{2}}\sum_{i=1}^{L}\left(i-1\right).$$

As the energy consumed in EDS depends on the number of attempt before broadcast, the energy consumed in EDS-S can be computed by placing the values of $n_{i}$ and $P_{ci}$ into (4)–(6) to obtain the following expression for cost.

(11) $$\begin{array}{cc} \beta (L) & =2\left(N-1\right)+L+\frac{Lt_{1}^{2}\pi }{6x^{2}}[\left(L^{2}-1\right)\left(N-1\right)\\ \; & +3(N-1)(L-1+2\pi )-6L]\\ \; & -\frac{\pi^{2}L^{2}t_{1}^{4}}{2x^{4}}\left(L-1\right)\left(N-1\right). \end{array}$$

The expression in (11) provides the expected broadcast cost for energy consumption of EDS-S as a function of the TTL value, the number of attempts before broadcast, and the network area. The maximum number of attempts required to search the entire network is represented by κ, i.e., $\kappa =\frac{x}{\sqrt{\pi t_{1}}}$ [30].

### 3.4. Increment TTL with Linear Function (EDS-L)

**Figure 6:**
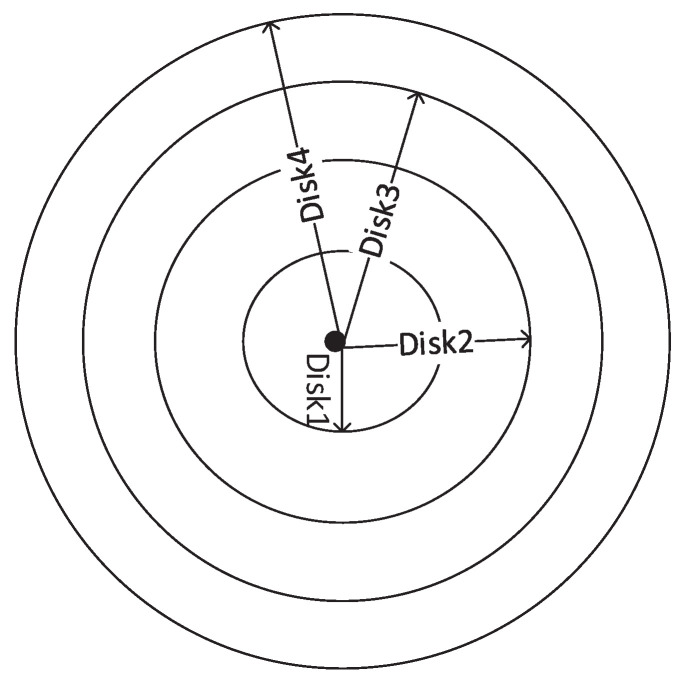
Disk Formation for EDS-L.

In this case, the TTL value is incremented such that the area of each CRB increases linearly with an increase in TTL value, as shown in Figure 6. As the area of proceeding CRB is greater than the previous, it accommodates more nodes, and the probability of finding the destination increases as the number of attempts increases. Mathematically, $A_{c2}=2A_{c1},A_{c3}=3A_{c1}$, and, in general, $A_{cn}=nA_{c1}$. Formulating in the similar pattern as in case of EDS-S, the TTL value required to search *i*th CRB is $t_{i}=it_{1}$. Then, the number of nodes in each CRB can be calculated as follows.

(12) $$n_{i}=\frac{\left(N-1\right)\left(2i-1\right)\pi t_{1}^{2}}{x^{2}}.$$

The probability that the destination lies in the *i*th CRB is given by the following.

(13) $$P_{ci}=\frac{\pi t_{1}^{2}\left(2i-1\right)}{x^{2}}.$$

Then, the expected time to find the destination can be calculated as follows.

(14) $$E\left(t\right)=\frac{2\pi t_{1}^{3}}{x^{2}}\sum_{i=1}^{L}i\left(2i-1\right)+\frac{T\pi t_{1}^{2}}{x^{2}}\sum_{i=2}^{L}\left(i-1\right)\left(2i-1\right).$$

The energy consumed during route discovery process can be calculated by placing the values of $P_{ci}$ and $n_{i}$ into (4)–(6), which provides X(L), Y(L), and Z(L), respectively, as follows

(15) $$\begin{array}{cc} X(L) & =\frac{L\pi t_{1}^{2}\left(L+1\right)\left(4L-1\right)}{6x^{2}}\\ \; & +\frac{L\pi t_{1}^{2}\left(L-1\right)\left(L^{2}-1\right)\left(4L^{2}-1\right)}{5x^{2}}, \end{array}$$

(16) $$Y\left(L\right)=L+N-1+\frac{L\left(L-1\right)\left(2L-1\right)\left(N-1\right)\pi t_{1}^{2}}{x^{2}},$$

(17) $$Z\left(L\right)=1-\frac{\pi t_{1}^{2}}{x^{2}},$$

Now, solving (3) provides the expected broadcast cost for EDS-L where the expected broadcast cost is a function of the ratio of TTL value and network area. The maximum number of attempt required to search entire network is $\kappa =\frac{2x}{t_{1}\sqrt{\pi }}-1$, where $t_{1}>\frac{2x}{\sqrt{\pi }}$.

### 3.5. Increment TTL with Recursive Function (EDS-R)

**Figure 7:**
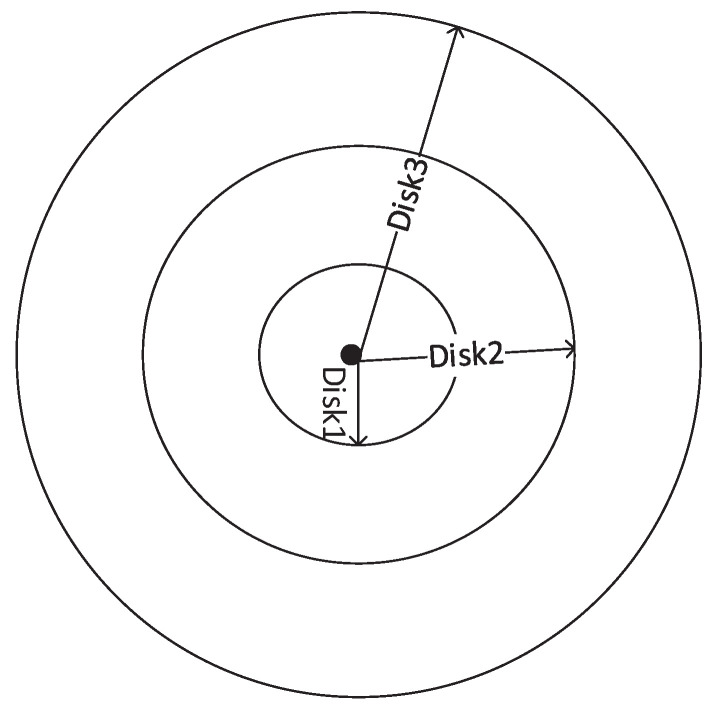
Disks Formation for EDS-R.

In this variant, the TTL value is chosen such that the area of each CRB increases non-linearly with an increase in TTL value as shown in Figure 7.

Mathematically, we have $A_{c2}=2A_{c1},A_{c3}=2A_{c2},A_{c4}=2A_{c3}$, and, in general, $A_{cn}=2A_{c(n-1)}$. Assuming that the area of each CRB increases and $t_{1}$ is the initial value of TTL set by the source node, then the TTL value required to search the *i*th disk is given by the following.

(18) $$t_{i}=\sqrt{\frac{i(i+1)}{2}}t_{1}.$$

The number of nodes in each CRB can be calculated as follows:

(19) $$n_{i}=\frac{\left(N-1\right)i\pi t_{1}^{2}}{x^{2}},$$

and $P_{ci}=\frac{i\pi t_{1}^{2}}{x^{2}}.$ The expected time to find the destination can be calculated by placing the values from (19) and (20) into (2), which provides the following.

(20) $$\begin{array}{c} E\left(t\right)=\frac{2\pi t_{1}^{3}}{x^{2}}\sum_{i=1}^{L}\left(i\sqrt{\frac{i(i+1)}{2}}\right)+\frac{T\pi t_{1}^{2}}{x^{2}}\sum_{i=2}^{L}i(i-1), \end{array}$$

As we assume that each node will transmit a single copy of the RREQ packet, the energy consumption of the route discovery is directly proportional to the number of nodes involved during the process. Then, solving (4)–(6) by using the values of $n_{i}$ and $P_{ci}$ yields the expected broadcast cost for EDS-R as follows.

(21) $$\begin{array}{cc} \beta (L) & =\frac{L(L+1)}{2}-\frac{\pi Lt_{1}^{2}\left[3L^{3}+L^{2}+6N\left(L+1\right)\right]}{12x^{2}}\\ \; & +\frac{\pi t_{1}^{4}\left[L^{2}{\left(L+1\right)}^{2}\left(2L+1\right)\right]}{24x^{4}}+N. \end{array}$$

The maximum number of attempts required to search the entire network is $\kappa =u-\frac{1}{2}$, where $u=v-\frac{a}{3v}$, $v={\left(\frac{-27b+\sqrt{729b^{2}+108a^{3}}}{2}\right)}^{\frac{1}{3}}$, $a=-\frac{13}{4},\;\mathrm{and}\;b=2-\frac{3x^{2}}{\pi t_{1}^{2}}$.

## 4. Experimental Results

We consider a wireless network setup with devices uniformly distributed in the square region. We implement multiple sources scenarios to evaluate the performance of the proposed scheme under heavy traffic load, congestion, long data queues, and high time delays. Moreover, the random waypoint mobility model implementation on each node incorporates the effect of topological changes on the performance of the proposed scheme introduced due to link breaks, active route time outs, and route errors, which makes the experimental scenario more practical to test the performance of the proposed algorithm. The theoretical expressions are plotted using Matlab, while simulations are performed using Omnet++ simulation software. EDS is implemented by modifying the source code of AODV available in Omnet++ library.

### 4.1. Experimental Setup

In the experimental setup, we randomly deployed 32 nodes in the network area of 600 × 600 m^2^. In order to test the performance of proposed scheme under heavy traffic load, half of the nodes are configured as sources and the remaining half are configured as destination nodes. Moreover, the number of nodes has been increased up to 100 in order to analyze the performance of the proposed technique under high density scenarios. In the case of high density networks, 50 nodes are configured as sources, and 50 are configured as destination nodes. All nodes are configured as ad hoc hosts to relay other nodes’ data alongside their own traffic. We consider that each node in the network has a wireless Network Interface Card (NIC), which implements physical and Medium Access Control (MAC) layer protocols.

The source nodes start their communication in the third time slot after the network is deployed. This provides enough time to send some *hello* packets to establish a connection with the neighbors. The maximum transmission power of the node is set to $p_{t}=$2 mW, and the transmission range of the node depends on the chosen value of receiver sensitivity and the threshold value of SINR. The source nodes generate data packets after every 0.2 s. When a source node desires to send data towards the destination node, it searches a route to the destination in its routing table. If an active route is not found, it buffers the data packets in its transmit buffer and initiates the route search process. The proposed EDS algorithm is embedded at the network layer of each node to establish routes between the nodes by modifying the source code of AODV in Omnet++. The results are analyzed and averaged for 20 iterations for different node densities and data rates. We used the state-of-the-art ERS technique as the benchmark for performance comparison. The simulation parameters are summarized in Table 4.

### 4.2. Results and Discussion

In this section, we present and discuss the results of the proposed algorithm for various network parameters and analyze the performance by comparing other route search methods.

**Figure 8:**
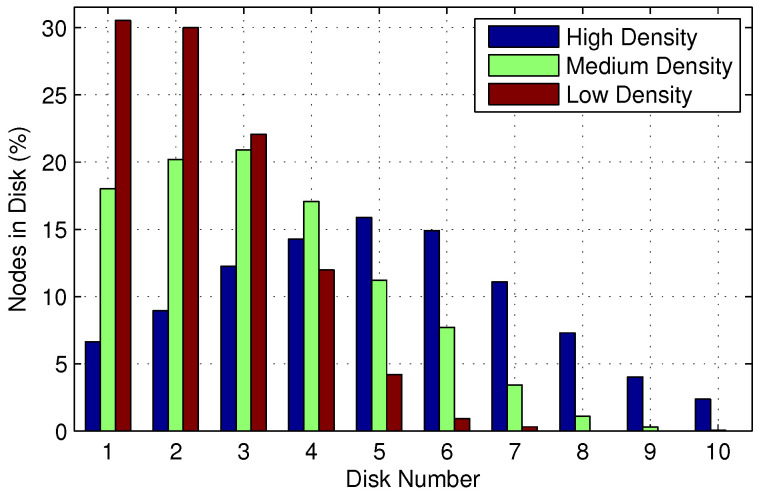
Nodes in the disk for EDS.

In Figure 8, we show the number of nodes in the disk with the number of iterations for low, medium, and high-density networks. In this setup, 100 nodes are uniformly distributed in the network area, with the source node in the center and the destination is chosen randomly. Figure 8 shows that the number of nodes in nearby disks to the source is high, and this number decreases in proceeding disks for low-density networks. For medium density networks, maximum nodes lie in two and three disks, whereas for high-density networks, the maximum nodes are accommodated in five to six disks. Figure 8 further shows that a higher value of TTL is required to accommodate maximum nodes in the disk when node density increases.

**Figure 9:**
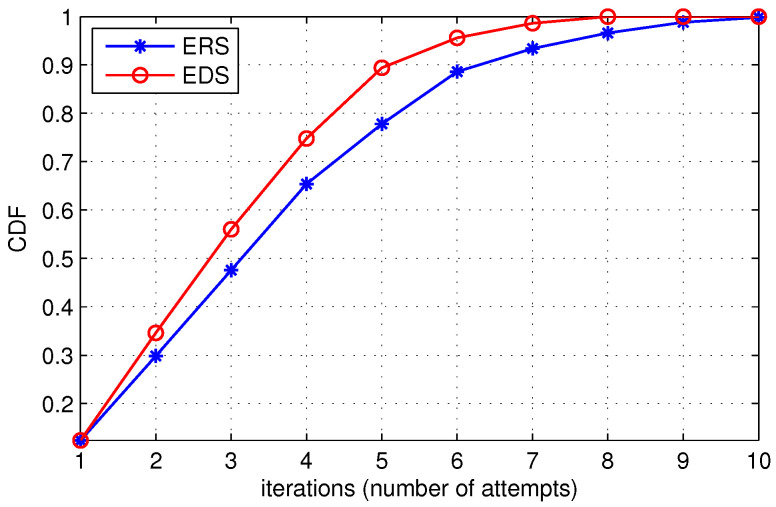
Probability to locate the destination.

In Figure 9, we show the Commutative Distribution Function (CDF) of the probability to find the destination with respect to the number of iterations or number of attempts made by the source node. The graph shows that the probability of finding the destination increases with an increase in the number of iterations. With the increase in the number of iterations, the search area around the source node increases, and more nodes are accommodated in the disk, increasing the probability of finding the destination. Figure 9 shows that the probability of finding the destination in the case of EDS increases at a higher rate as compared to ERS. For instance, ten iterations are needed to locate the destination in ERS, but EDS can locate the destination in eight iterations. Hence, locating the destination in fewer iterations saves waiting time as well as reduces cost.

**Figure 10:**
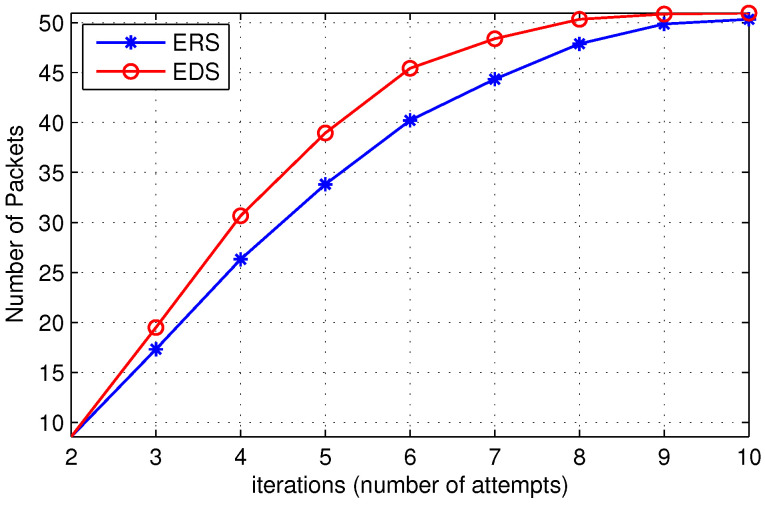
Cost of a single search query.

**Figure 11:**
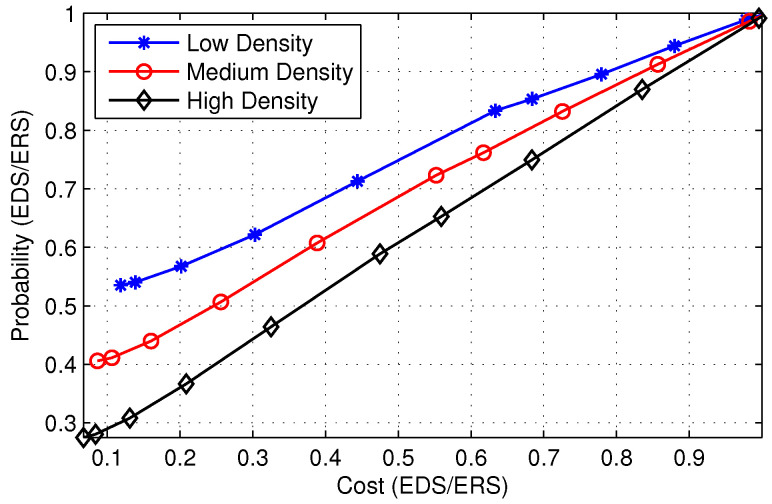
Comparison of ERS and EDS.

In Figure 10, we show the cost of a single search query in the destination search process with respect to the number of iterations. Figure 10 shows that the cost of EDS is slightly higher than ERS. However, (2) demonstrates that the average search cost of EDS is lower as compared to ERS due to the high probability of finding the destination. Figure 11 further elaborates the point where we plot the ratio of probability to find the destination and cost of the search for ERS and EDS algorithms. As the slope of the line is greater than one, it implies the probability of finding the destination in case of EDS increases at a higher rate than the increase in cost. Figure 10 further illustrates that the slope of the line decreases as node density in the network increases. This trend is observed because the exponential increase in cost overcomes the beneficial probability factor when node density is high. Therefore, the performance of EDS is almost the same as ERS if node density is high.

**Figure 12:**
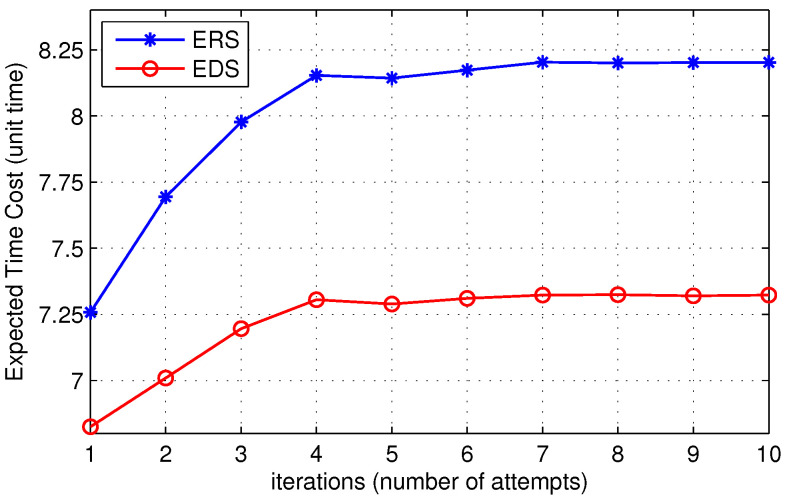
Time cost of ERS and EDS.

**Figure 13:**
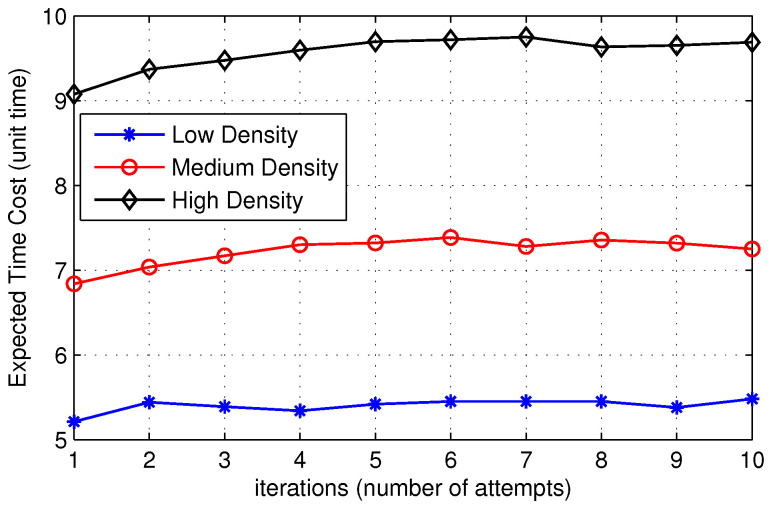
Comparison of time cost of EDS for various network densities.

In Figure 12, we show the expected time to find the destination with respect to the number of iterations. The x-axis represents the threshold value of TTL chosen by the source node while initializing the route discovery process. In other words, it represents the number of attempts made by the source node to search the destination node by expanding the search area before it decides to initiates a broadcast packet. Figure 12 shows that the expected time to locate the destination in the case of EDS is lower than ERS. The proposed solution accommodates more nodes in the search area as compared to the ERS algorithm. As a result, the probability of finding the destination increases, reducing the expected time to find the destination. Furthermore, in Figure 13, we show the expected time to find the destination for low, medium, and high-density networks. Figure 13 shows that the expected time to locate the destination is short for low-density networks. When the number of nodes in the network increases, the average number of hops between the source and the destination increases, which increases the expected time to find the destination.

**Figure 14:**
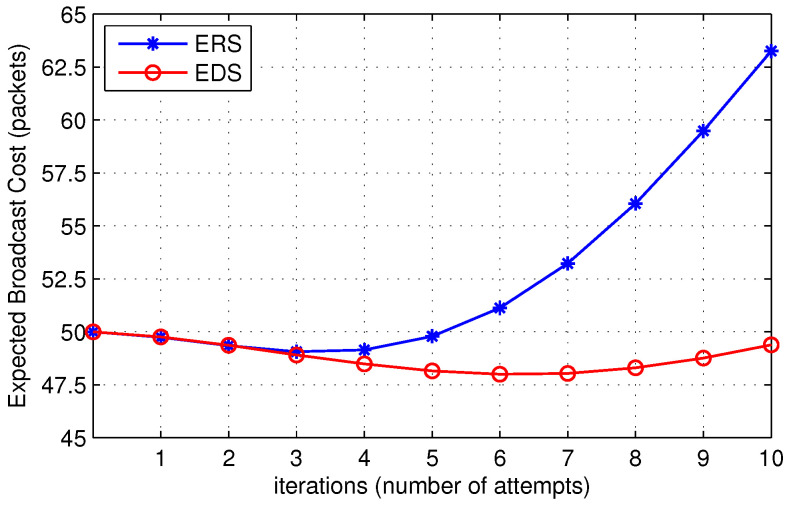
Expected broadcast cost for route discovery.

Figure 14 shows the expected broadcast cost with respect to the number of iterations or number of attempts before broadcast. It can be observed from the figure that the expected broadcast cost of EDS and ERS is almost the same at a low value of iterations. If the destination is not far away from the source node, the number of nodes in the ring and disks is almost the same; thus, broadcast cost is the same at a low value of iterations. However, when the distance between the source and the destination increases, the expected broadcast cost of EDS is much lower than ERS. The expected broadcast cost follows a decreasing trend up to five to six iterations. It then increases with an increase in iterations, which shows an optimum value of the number of attempts that provides the minimum cost. Figure 14 also shows that the source node can achieve the lowest cost if it chooses a threshold value between four and six number of attempts. Increasing the threshold above six increases the cost of failure, which results in high expected broadcast cost.

**Figure 15:**
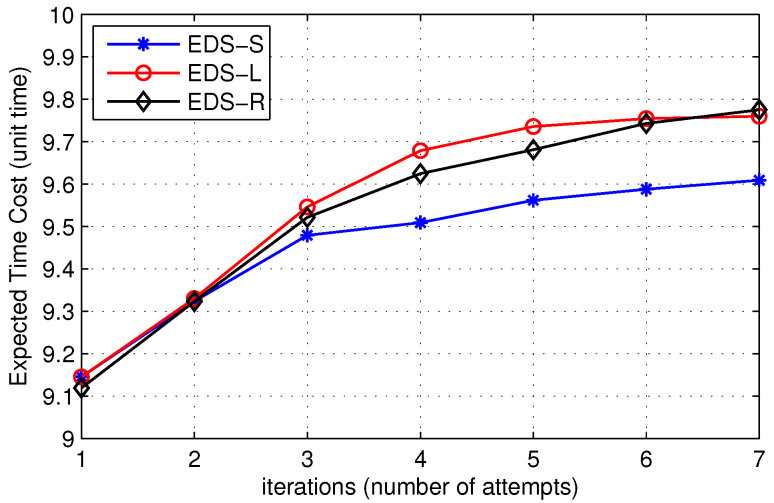
Effect on expected time to locate the destination for different TTL choices.

Figure 15 shows a comparison of the expected time to locate the destination for three variants of the proposed EDS. Figure 15 compares the expected time to locate the destination for EDS-S, EDS-L, and EDS-R where time latency of route discovery increases with the number of iterations. Figure 15 further shows that the expected time to find the destination of EDS-S is lower than EDS-L and EDS-R for a high number of iterations. On the other hand, at a low number of iterations, the destination search time of EDS-R is less than the other two variants. The time latency of all the techniques is almost the same at low TTL values, but the performance of EDS-S is better than the other two when the distance between the source and the destination is relatively high.

**Figure 16:**
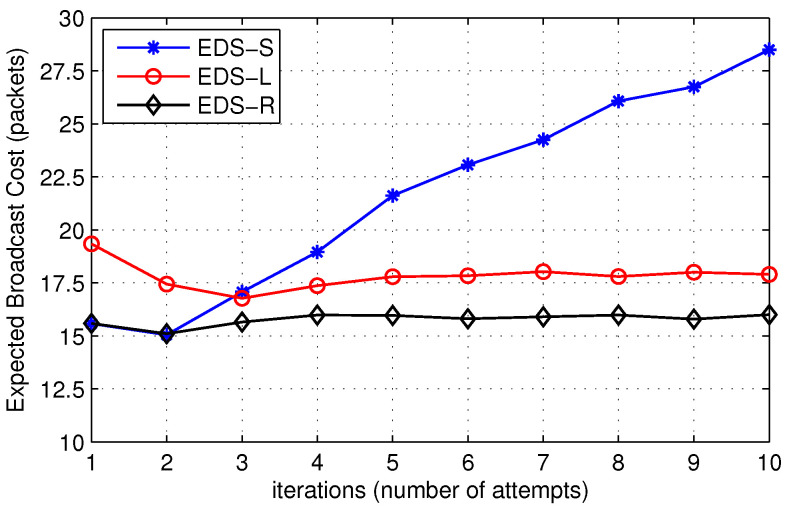
Effect on expected broadcast cost for different TTL choices.

In Figure 16, we show the expected broadcast cost of EDS-S, EDS-L, and EDS-R. It is clear that the broadcast cost initially decreases up to some iteration then follows an increasing trend. Initially, when the disk size is small, the number of packets relayed in the network and the probability of finding the destination in the disk are not high enough. When the source node increases its TTL value or equivalently the disk area, the probability of finding the destination increases, which reduces the number of network-wide broadcasts, resulting in a decrease in the overall search cost. Furthermore, an increase in the number of iterations increases the cost of expanding search procedure, which sometimes exceeds the network-wide broadcast cost. Thus, there is an optimal value for the number of iterations between low and high values that provides minimum route search cost. Moreover, Figure 15 and Figure 16 show that there is a trade-off between expected broadcast cost and expected time cost for the three techniques. The expected time cost of EDS-S is lower than the other two, but its expected broadcast cost is high. Similarly, EDS-R performs well in terms of expected broadcast cost, but its expected time to locate the destination is higher than EDS-S. Therefore, we can conclude that EDS-S is a suitable choice when reduced time latency is a major requirement. Similarly, EDS-R is appropriate to implement in situations where nodes do not have enough energy resources, such as IoT networks in 5G.

**Figure 17:**
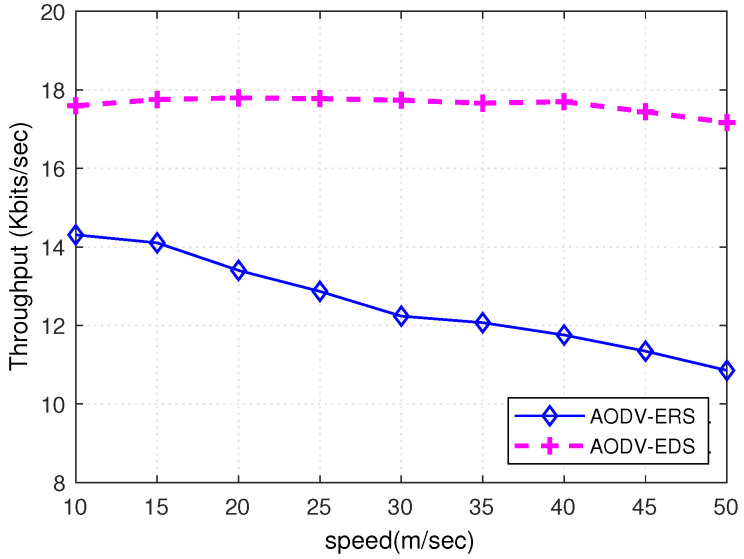
Throughput Comparison of ERS and EDS.

**Figure 18:**
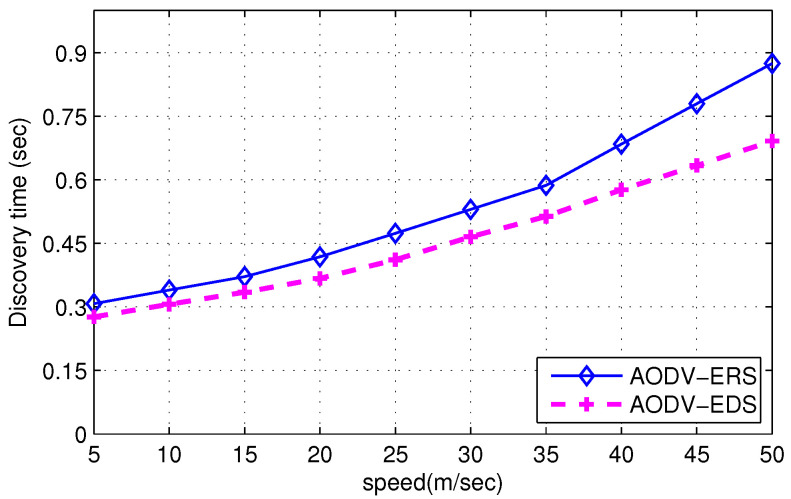
Latency Comparison of ERS and EDS.

Figure 17 shows the throughput comparison of ERS and EDS algorithms when implemented on the AODV routing protocol. Figure 17 shows that the proposed EDS technique outperforms the conventional ERS scheme; when the mobility of nodes increases, throughput decreases. The rate of decrease in throughput is much lower when EDS is used as a destination search algorithm. The EDS algorithm searches the destination in a lower number of attempts, which reduces time delays and length of the queue at the nodes, resulting in high throughput. Furthermore, Figure 18 shows the time to search the destination of ERS and EDS techniques with respect to node mobility. Figure 18 shows that the time to find the destination of EDS is lower than the ERS algorithm. However, the time to find the destination increases with an increase in node mobility; when a node moves at high speed, the number of disconnections between nodes increases, increasing the time to find the destination. Nevertheless, EDS uses expanding disk-like pattern to search the destination, decreasing search time compared to ERS.

Table 5 summarizes performance comparison of the proposed method with Expanding Ring Search (ERS), Distribute Weighted Clustering Algorithm (DWCA), and Cluster Head based Modified-Blocking Expanding Ring Search (CMBERS+) algorithms. The comparison shows that the proposed search method outperforms previously proposed schemes in terms of high network throughput and less route latency.

## 5. Conclusions

This paper proposes a destination search routing method for distributed 5G and beyond networks. This work shows that the EDS algorithm performs better as compared to the previously proposed algorithm in terms of reduced route latency, low energy consumption, high network throughput, and minimum routing overhead. The results shows an improvement of 12% in terms of route latency, 48% higher network throughput, and 62% reduced routing overhead as compared to ERS-based algorithms. The improvement becomes evident in the scenarios when node speed increases and topological changes occur frequently. Moreover, our analysis shows that there exists a time-cost trade-off between the three variants (EDS-L, EDS-S, and EDS-R) presented for the destination search problem. The choice of solution depends on the requirement of the user and network parameters. 

## Figures and Tables

**Table 1:** Summary of Related Work.

Proposed Method	Findings	Limitations
BKNS [14]	Selects the neighbour with largest node degree to accelerate the destination search	Only suitable when source and destination are in close vicinity. Periodic update of neighbour degree is required for dynamic networks such as Vehicle-to-vehicle.
Distributed Routing [15]	Waiting time as a function of network density is used to reduce number of rebroadcast of RREQ packets.	Network density is a global parameter that is difficult to calculate in distributed networks.
Congestion Controlled Routing [16]	Calculate the congestion on the node before taking routing decision.	Not suitable for mobile networks as traffic load changes with network topology.
Probabilistic Broadcast [18]	Each node broadcast packet with a predefined probability to reduce number of rebroadcasts.	Increased hop count and end-to-end time latency.
BERS [23]	Waiting time as function of hop count to reduce RREQ packets.	High time latency.
DWCA [24]	Cluster based routing to limit the search region.	The child node of neighboring cluster head may continue propagation of RREQ even after destination is found.
CMBERS+ [25]	Improves DWCA by introducing chase packets to reduce over propagation of RREQ.	Frequent adaptation of cluster head is required to fair distribution of management operations.

**Table 2:** Area of CRB and Disks.

Disk Number	Radius	TTL	Area of Disk	Area of CRB
1	$r_{1}$	$t_{1}$	$\pi r_{1}^{2}$	$\pi (r_{1}^{2}-r_{0}^{2})$
2	$r_{2}$	$t_{2}$	$\pi r_{2}^{2}$	$\pi (r_{2}^{2}-r_{1}^{2})$
3	$r_{3}$	$t_{3}$	$\pi r_{3}^{2}$	$\pi (r_{3}^{2}-r_{2}^{2})$

**Table 3:** Disk Access Time.

Disk Number	TTL	Waiting Time	Disk Access Time
1	$t_{1}$	0	0
2	$t_{2}$	*T*	*T*
3	$t_{3}$	*T*	2T
4	$t_{4}$	*T*	3T

**Table 4:** Simulation Parameters.

Parameter Name	Value
Network Parameters	
Network area	600 × 600 m^2^
Number of nodes	32–100
Number of sources	16
Number of destinations	16
Node Parameters	
Battery	12 V/3800 mAh
Traffic source	CBR
Message length	512 B
Send interval	0.2 s
Transmission start time	3 s
Frame capacity	100
Maximum queue size	14
RTS threshold bytes	3000 B
MAC retry limit	7
MAC header length	10 B
Queue type	FIFO
Maximum transmission power	2 mW
Radio sensitivity	−85 dBm
Radio SINR threshold	4 dBm
Broadcast delay	uniform (0 s, 0.005 s)
Mobility	Random waypoint mobility
Wait time	uniform (0 s, 1 s)
Routing Protocol Parameters	
Hello Interval	0.5 s
Maximum periodic jitter	0.125 s
Active route timeout	3 s
TTL start	0.08 s
TTL threshold	0.54 s
Maximum allowed TTL	2 s
Node traversal time	0.04 s
RREQ buffer time	0.32 s

**Table 5:** Performance comparison of EDS with other route discovery algorithms.

Technique	EBC (%)	Throughput (%)	Average Latency (s)
ERS	49.8–62.5	25–48.97	0.30–0.78
DWCA	30.1–53.2	2.29–5.79	0.25–1.90
CMBERS+	42.56–83.2	12.04–25	0.20–1.80
